# Medicine Goes to School: Teachers as Sickness Brokers for ADHD

**DOI:** 10.1371/journal.pmed.0030182

**Published:** 2006-04-11

**Authors:** Christine B Phillips

## Abstract

Christine Phillips explores the roles of teachers as brokers for ADHD and its treatment, and the strategies used by the pharmaceutical industry to frame educators' responses to ADHD.


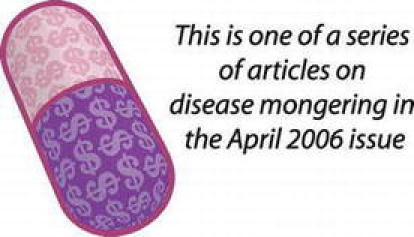


Over the last twenty years, attention deficit hyperactivity disorder (ADHD) has emerged as a disorder of importance in childhood. Prescription of psychostimulants for ADHD escalated in many countries through the 1990s. Between 1990 and 1995, prescriptions of methylphenidate for young people increased 2.5-fold in the US [ 1], and 5-fold in Canada [ 2]. In New South Wales, Australia, rates of treatment for children in 2000 were nine times those in 1990 [ 3].

ADHD joins dyslexia and glue ear as disorders that are considered significant primarily because of their effects on educational performance. Medicalising educational performance can help children receive specialised medical and educational services; at the same time it can lead to them receiving medications or surgical therapies which may have short-term and long-term ill effects.

In the case of ADHD, there has been a complex, often heated debate in the public domain about the verity of the illness and the personal cost-benefit ratio of treatment with psychostimulant medication [ 4–6]. Much of the polemic for and against psychostimulants is concerned with the part played by doctors, the prescribers of medication, in diagnosing or discounting ADHD. ADHD is, however, a disorder of educational performance, and so teachers have a critical role in advocating for the illness, and its medical treatment. This essay explores the roles of teachers as brokers for ADHD and its treatment, and the strategies used by the pharmaceutical industry to frame educators' responses to ADHD.

## The Teacher's Role in Managing ADHD

In his essay on medicalisation processes, Conrad argued that when disorders previously viewed as non-medical are redefined as sicknesses, non-medical people often perform the “everyday routine work” of disseminating understanding of the new sickness [ 7]. A temperance society worker, for example, might have disseminated the concept of alcoholism as a disease through everyday contacts with alcoholics and their families. With ADHD, the teacher's work extends beyond simply ensuring the disorder is understood by parents. Instead, the teacher participates in the diagnosis, and may broker different forms of treatment, or rejection of treatment. Brokerage is not a disinterested activity: teachers may have a vested interest in detecting and managing disruptive children, or they may adhere to beliefs about learning disorders which lead them to dissuade parents of the need for treatment.

The role of the teacher as the sickness and treatment broker for ADHD has been elaborated more clearly for ADHD than for any other childhood disorder. The DSM-IV diagnostic criteria accord teachers a formal role in diagnosis through specialised assessment instruments such as the Conners Teacher's Rating Scale [ 8]. Teachers often agree to administer psychostimulant medication during the school day, although there is in Australia, the UK, and the US no legal compulsion to do so. A subtle incentive for teachers to administer medication in the middle of the day may be the assurance of a tractable child in the afternoon.

An informal role also exists for teachers as “disease-spotters.” There appears to be considerable difference internationally in the alacrity with which teachers engage in disease-spotting. In a study of 491 physicians in Washington, D. C., almost half of the diagnoses of ADHD in their patients had been suggested first by teachers [ 9]. In the UK, on the other hand, parental concerns that a child has ADHD may be discounted by teachers [ 10].

## How Drug Companies Influence Teachers

As teachers have some agency in diagnosing ADHD, and may in fact contest the diagnosis, the pharmaceutical industry has an interest in directing teachers toward medical treatment. Pharmaceutical companies have been able to exploit the Internet to access teachers and to influence their brokerage role. The approach to teachers tends to mirror strategies used to familiarise doctors with pharmaceuticals.

### The pharmaceutical company as disinterested purveyor of education

The drug promotion that masquerades as professional education is such a fixture in the medical domain that many universities train medical students to critique promotional material. Both Shire (manufacturer of Adderell) [ 11] and Novartis (manufacturer of Ritalin) [ 12] have established educational websites separate from their own industry sites, each of which contains specific resources for teachers. On a page entitled “If parents ask…,” Novartis suggests responses teachers might make to concerned parents:

“Make it clear to them that it is important for them—and their child—to understand and follow the doctor's medical advice about medication and other therapies for ADHD. ADHD is a serious condition that may require the child to be on medication and undergo counselling for a long duration [ 12].”

Each site incorporates links to the manufacturer responsible for the site [ 13] or directly to the psychostimulant produced by the manufacturer [ 14], discussion of the diagnostic process, and references to the legislation governing the rights of access for disabled people to treatment, such as the Individuals with Disabilities Education Act in the US. An activity such as Shire's funding of an annual toll-free “ask the experts” ADHD hotline, 1-888-ASK-ADHD, [ 15] is another example of the provision of advertorial information to teachers in the guise of objective education. Experts provided for this free hotline, now in its seventh year, include teachers, as well as school nurses, doctors, and advocates; suggested topics include the management of ADHD within the school.

Other school personnel are also targeted. In 1997, Novartis collaborated with the National Association of School Nurses in the US to run a nationwide campaign, in which 11,000 school nurses were provided with a resource kit containing information on ADHD, its treatment, and various support organisations [ 16]. Novartis later collaborated with the National Association of School Nurses and others to produce a resource aimed at curbing misuse of psychostimulant medication, which again provided links to Novartis pharmaceuticals [ 17].

### Support of advocacy groups which lobby teachers

In the US, the pre-eminent advocacy group for people with ADHD is CHADD (Children and Adults with Attention Deficit/Hyperactivity Disorder). In the 2004–2005 financial year (year ended 30 June 2005), 22% of CHADD's total revenue came from the pharmaceutical industry [ 16]. CHADD undertakes educational programs for teachers [ 18], including acting as the lead editorial consultant of a special issue on ADHD in Health in Action, a quarterly publication of the American School Health Association [ 19]. The UK's answer to CHADD, the National Attention Deficit Disorder Information and Support Service (ADDISS), also carries a brief to develop and publicise educational programs for teachers. A charity-based organisation set up by the Department of Health, the service has also received funding from Janssen-Cilag, UCB Pharma, and Eli Lilley, according to reports in the UK press [ 20].

### Creating a presence in the school for the pharmaceutical industry

A more general staking of claim to a role in schools is provided through the range of online science educational materials now provided by GlaxoSmithKline [ 21], Pfizer [ 22], and the Association of the British Pharmaceutical Industry [ 23]. Although these sites do not mention specific medications, they reinforce the place of the pharmaceutical industry as a benevolent and authoritative presence within the school, much as the provision of branded educational materials to doctors reinforces the position of the pharmaceutical industry within the clinic.

## Conclusion

The organised penetration of the pharmaceutical industry associated with ADHD into the education domain is a new phenomenon. While there has been extensive discussion about the ethics of fast-food marketing within schools [ 24, 25], there has been little about the consequences of the pharmaceutical industry's infiltration of schools.

It could be argued that in providing information to teachers, pharmaceutical industries are simply acting as good corporate citizens. Such an argument would carry more weight if these companies also provided education programs addressing autism and dyslexia, two other conditions which impact upon educational performance, but which do not have accepted pharmaceutical therapies. While there is an argument for providing unbiased education to teachers about a high-profile condition, education provided by pharmaceutical companies is self-serving in that it often provides education which references their own products, and channels the reader toward medical therapy.

There are calls for doctors to learn about pharmaceutical marketing strategies in their training [ 26, 27], to participate in the monitoring of outcomes of medication, through post-marketing surveillance, and to maintain a global watch on pharmaceutical marketing [ 28]. The wide acceptance of disorders of educational performance, and the penetration of the pharmaceutical industry into schools, point to similar needs for teacher training and participation in surveillance (see Box 1). Children have no agency in this market. To be effective advocates for children, teachers need to be supported to be objective and accurate interpreters of information for parents and healthworkers, rather than franchisees in the sickness marketplace. 3

**Box 1. Suggestions to Support Teachers as Independent Advocates for Children with ADHD**

- Teachers should be trained to decode and question marketing strategies used by the pharmaceutical industry, just as medical students are.
- Teachers should have a mechanism to report their observations about medication to an independent body, such as Australia's Adverse Drugs Reaction Advisory Committee.
- Teachers should contribute to documenting educational and other outcomes of children with ADHD, through participating in formal collation of data across school regions about outcomes.

## 

**Figure:**
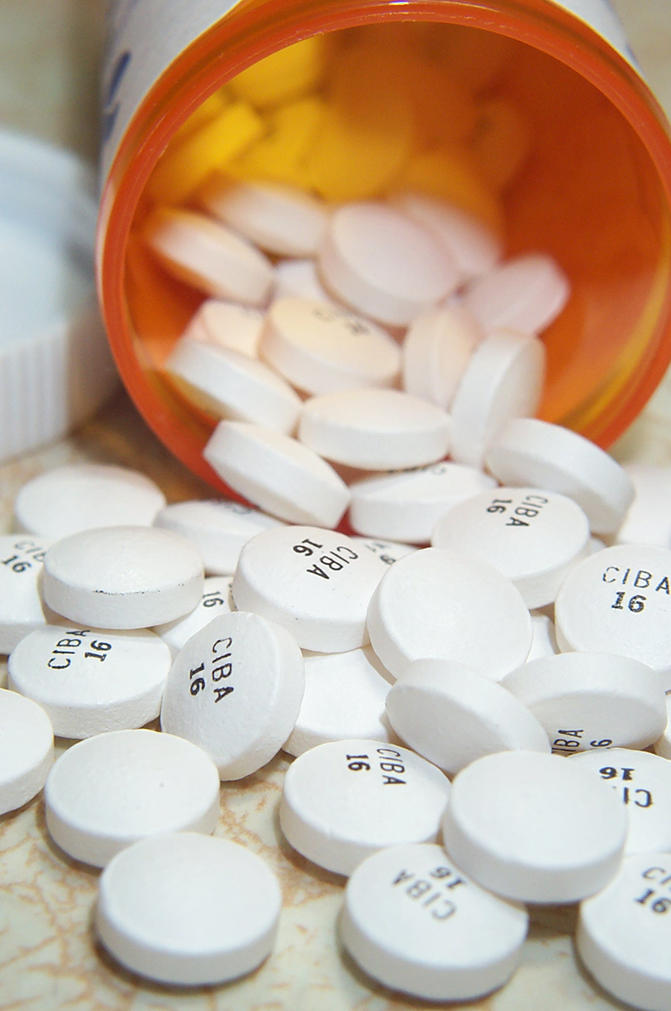
Prescription of psychostimulants for ADHD escalated in many countries through the 1990s (Photo: Sponge, published under the GNU Free Documentation License)

## Abbreviations

ADHD: attention deficit hyperactivity disorder
CHADD: Children and Adults with Attention Deficit/Hyperactivity Disorder
